# Supplementary material for: Access to HIV Antiretroviral Therapy among People Living with HIV in Melbourne during the COVID-19 Pandemic
Source: Int J Environ Res Public Health. 2021 Dec 3;18(23):12765. doi: 10.3390/ijerph182312765 (PMC8657228; doi:10.3390/ijerph182312765)
Supplement: Supplementary file 1 [file ijerph-18-12765-s001.zip › Figure S1.pdf]

Cost centre	ID	Dispense date	Product	Dispense net pack quantity
GP Clinic	1	20/02/2018	Abacavir-Lamivudine	4 packs
GP Clinic	1	20/02/2018	Darunavir-Cobicistat	4 packs
GP Clinic	1	20/02/2018	Dolutegravir	4 packs
GP Clinic	2	20/06/2018	Elvitegravir-Cobicistat-Emtricitabine-Tenofovir Alafenamide	2 packs
GP Clinic	2	13/08/2018	Elvitegravir-Cobicistat-Emtricitabine-Tenofovir Alafenamide	2 packs
GP Clinic	2	13/08/2018	Elvitegravir-Cobicistat-Emtricitabine-Tenofovir Alafenamide	2 packs

↓

	ID	Dispense date	Product	Dispense net pack quantity
GP Clinic	1	20/02/2018	Abacavir-Lamivudine<br>Darunavir-Cobicistat<br>Dolutegravir	4 packs
GP Clinic	2	20/06/2018	Elvitegravir-Cobicistat-Emtricitabine-Tenofovir Alafenamide	2 packs
GP Clinic	2	13/08/2018	Elvitegravir-Cobicistat-Emtricitabine-Tenofovir Alafenamide	4 packs

Figure S1. An example of the data cleaning process demonstrating combining multiple drugs recorded on the same date. If the drugs are different, they are combined as a single combination therapy. If the drugs are the same, they are combined as a single dispensing record and the dispense net pack quantity is multiplied.
