# Supplementary material for: Access to HIV Antiretroviral Therapy among People Living with HIV in Melbourne during the COVID-19 Pandemic
Source: Int J Environ Res Public Health. 2021 Dec 3;18(23):12765. doi: 10.3390/ijerph182312765 (PMC8657228; doi:10.3390/ijerph182312765)
Supplement: Supplementary file 1 [file ijerph-18-12765-s001.zip › Table S1.pdf]

Table S1. Medication Possession Ratios (Alternative approach)

GP and MSHC patients	Years	Mean	Standard deviation	Median	IQR
	2018	0·92	0·24	0·99	0·82 – 0·99
	2019	0·94	0·23	0·99	0·82 – 0·99
	2020	0·92	0·25	0·99	0·82 – 0·99

GP = general practice; IQR = Interquartile range; MSHC = Melbourne Sexual Health Centre

Alternative approach = MPR calculated by the total number of days covered by every ART script in a year divided by 365
