# Supplementary material for: Access to HIV Antiretroviral Therapy among People Living with HIV in Melbourne during the COVID-19 Pandemic
Source: Int J Environ Res Public Health. 2021 Dec 3;18(23):12765. doi: 10.3390/ijerph182312765 (PMC8657228; doi:10.3390/ijerph182312765)
Supplement: Supplementary file 1 [file ijerph-18-12765-s001.zip › Table S2.pdf]

Table S2. Medication Possession Ratios for individuals with data for both 2019 and 2020 (*N*=2454)

GP and MSHC patients	Years	Mean (SD)	95% Confidence interval	Median	IQR
	2018	1·05 (1.81)	0·99 to 1·11	0·99	0·91 – 1·07
	2019	1·07 (2.14)	0·99 to 1·14	0·99	0·91 – 1·07
	2020	1·13 (3.91)	1·00 to 1·26	0·99	0·90 – 1·07

GP = general practice; IQR = Interquartile range; MSHC = Melbourne Sexual Health Centre
