# Supplementary figures and images for: Access to HIV Antiretroviral Therapy among People Living with HIV in Melbourne during the COVID-19 Pandemic
Source: Int J Environ Res Public Health. 2021 Dec 3;18(23):12765. doi: 10.3390/ijerph182312765 (PMC8657228; doi:10.3390/ijerph182312765)

Weekly number of COVID-19 cases in Victoria

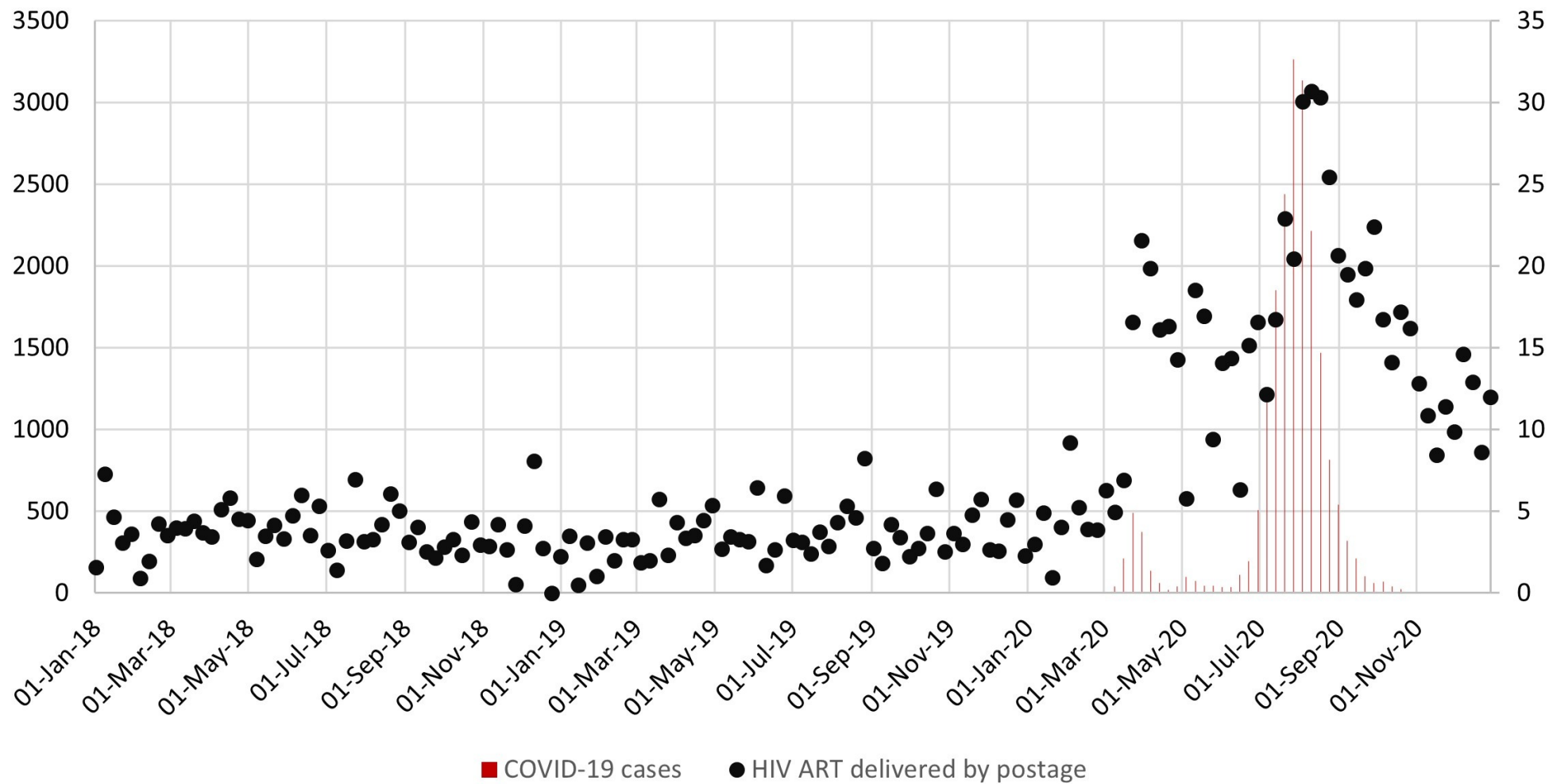

The proportion of HIV ART delivered by postage (%)

Supplement: Supplementary file 1 [file ijerph-18-12765-s001.zip › Figure S2.pdf]

Proportion of PLHIV  
with controlled viral load (%)

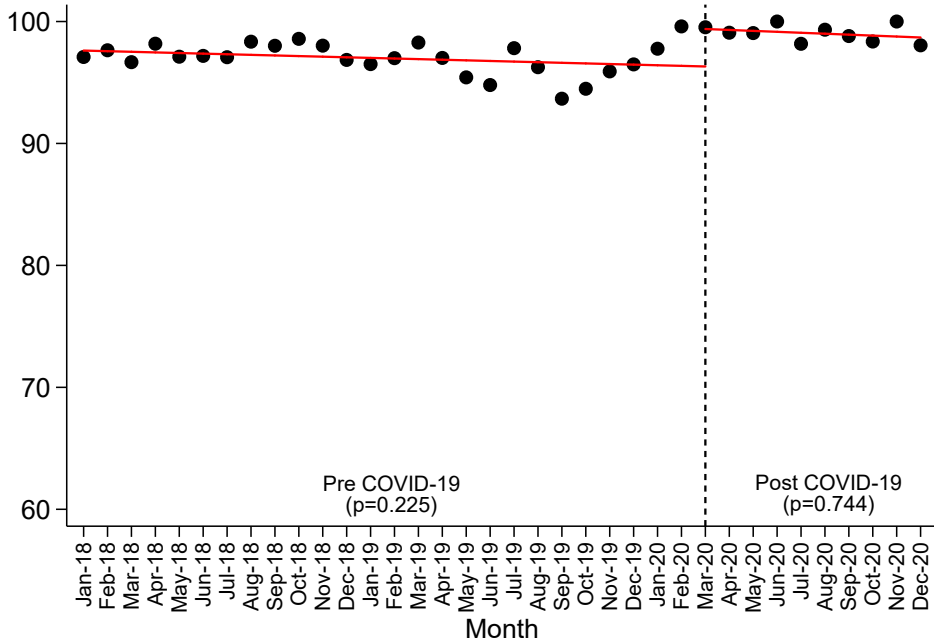

● Actual

— Predicted

Supplement: Supplementary file 1 [file ijerph-18-12765-s001.zip › Figure S3.pdf]
